# Folate Content and Yolk Color of Hen Eggs from Different Farming Systems

**DOI:** 10.3390/molecules26041034

**Published:** 2021-02-16

**Authors:** Marta Czarnowska-Kujawska, Anna Draszanowska, Elżbieta Gujska, Joanna Klepacka, Marta Kasińska

**Affiliations:** 1 Department of Commodity and Food Analysis, The Faculty of Food Sciences, University of Warmia and Mazury in Olsztyn, 10-719 Olsztyn, Poland; elka@uwm.edu.pl (E.G.); klepak@uwm.edu.pl (J.K.); emma3112@o2.pl (M.K.); 2 Department of Human Nutrition, The Faculty of Food Sciences, University of Warmia and Mazury in Olsztyn, 10-719 Olsztyn, Poland; anna.draszanowska@uwm.edu.pl

**Keywords:** folates, vitamins, food folate analysis, organic food, functional foods, HPLC, color analysis, yolk color

## Abstract

This study aimed to compare folate contents in hen eggs from four different farming systems, namely organic, free range, barn, and cage one. Folate retention during egg boiling was studied as well. The contents of individual folate vitamers were determined using the high-performance liquid chromatography method (HPLC), following trienzyme treatment. Folate content in eggs differed significantly (*p* < 0.05) due to the rearing system, with the highest mean content determined in the eggs from organic farming (113.8 µg/100 g). According to this study, one egg (60 g) may provide 40–86 µg of folates, which corresponds to 10–22% of the recommended daily intake for adults, 400 µg according to the Nutrition Standards for the Polish Population. The predominant folate form found in egg was 5-methyltetrahydrofolate, which showed considerably greater stability under boiling compared to 10-formylfolic acid present in a lower amount. In most eggs tested, the losses in total folate content did not exceed 15%. The color of yolk of the most folate-abundant organic eggs, had the highest value of lightness (L*) and the lowest value of redness (a*). This, however, does not correspond to consumer preferences of intense golden yolk color.

## 1. Introduction

**Figure 1:**
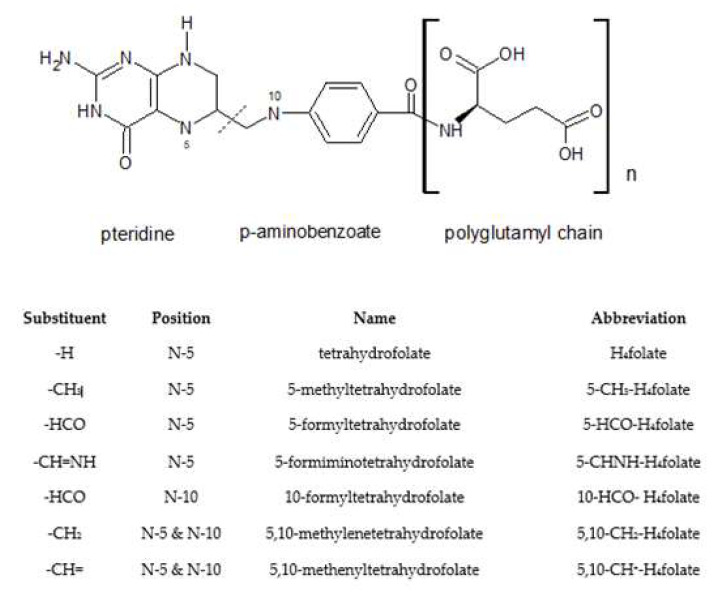
Structure of folic acid and polyglutamyltetrahydrofolates.

Folate (Figure 1) is the general term used for various forms of B-vitamins naturally occurring in foods and differing in the one-carbon substituent linked to nitrogen 5 or 10, the oxidative state of the pteridine ring, and the length of the glutamate chain, such as folic acid, 5-methyltetrahydrofolate, 5-formyltetrahydrofolate, tetrahydrofolate, 10-formylfolic acid, 5,10-methenyltetrahydrofolate, and 10-formyldihydrofolic acid [1,2,3]. Pure folic acid, the most oxidized folate form, is not usually found in food but because of the highest stability to chemical degradation and bioavailability it is used in vitamin supplements and fortified food products [4,5]. In humans, folate plays a key role in one-carbon metabolism, especially as a donor of the methyl group during DNA synthesis, metabolism of other vitamins (such as B2, B6, and B12) and certain amino acids (methionine, homocysteine) [6,7]. Humans cannot synthesize folates, thus, these vitamins need to be delivered with the diet. Although they are widely spread in the nature, their deficiency is common in both developed and undeveloped countries and can cause severe health problems [8], like the most well-known macrocytic or megaloblastic anemia [9]. Perhaps most widely publicized is folate contribution in reducing the risk of development of neural tube defects, such as spina bifida and anencephaly. Folates are also implicated in preventing other congenital anomalies and low birthweight [10]. The optimal folate status is important not only for women of childbearing age but also for the general population because of its role in the primary prevention of certain cancers, like colorectal [11,12], as well as neurocognitive decline in the elderly [13]. Some studies have indicated the association between a reduced risk of cardiovascular disease and stroke and a high folate intake or a high folate blood concentration [14,15].

Several strategies for increasing the folate intake can be distinguished. These are: (1) dietary supplementation with folic acid (most common in the pregnant and lactating women group); (2) the consumption of foods fortified, both obligatorily and voluntarily, with folic acid (cereal-based products such as flour, bread, pasta, breakfast cereals, fruit and vegetable juices, and others available commercially); and (3) increased consumption of foods naturally rich in folates. In 1998, the US and Canadian governments were the first ones to introduce a mandatory fortification of flours and cereal derivatives with folic acid. The obligatory fortification of flour resulted in the substantial improvement in folate and homocysteine status, followed by neural tube defects incidence reduction even up to 78%. Since then, almost 86 countries adopted this strategy [5,16,17]. However, due to health risks posed by the chronic exposure of the entire population to the synthetic form of the vitamin (folic acid), which can lead to the presence of unmetabolized folic acid in the organism, whose role remains unknown, no obligation to fortify food with folic acid has been introduced in any European country. The most frequently used argument against the regulation is the possibility of masking the macrocytic anemia of vitamin B12 deficiency in the elderly consuming high doses of folic acid [18,19,20,21]. Another reason for not choosing the mandatory folic acid fortification is the failure of clinical trials to prove its expected additional health benefits for the general population and not just for women of the childbearing age before conception. In the past years, no substantial reduction in the prevalence of neural tube defects has been observed in Europe, since there is no obligation to fortify foods with folic acid and many women do not follow the recommendation of diet supplementation with this vitamin in the periconceptional period [10]. That is why, it is highly important to encourage the consumption of foods that are naturally rich in folates.

Considering a high folate concentration, its most common sources include vegetables (especially the green ones), legumes, and certain fruits, whereas considering a high frequency of consumption of folate-rich products-it can be delivered with cereal products [8,22,23,24,25,26]. Less approved are folate-rich products of animal origin, such as liver and egg yolk. Their lower popularity may result from the former recommendation to eliminate saturated fats of animal origin from the diet to reduce the risk of cardiovascular disease development. Current studies indicate, however, that cholesterol and saturated fats should not be completely eliminated, and that, for instance, egg consumption can be safely increased up to 14 per week with no favorable effect on plasma LDL cholesterol [27].

Nowadays, the nutritional value of eggs is appreciated. Eggs are considered as a functional food, i.e., a significant source of macro and micronutrients, high-quality proteins, vitamins, and lipids, including polyunsaturated fatty acids (PUFA), and phospholipids. Health beneficials, including antioxidant properties, also result from the presence of: egg proteins (i.e., ovalbumin, ovotransferrin, in its native form or as hydrolytic pep-tides, ovomucoid and ovomucoid hydrolysates, phosvitin), selenium, and carotenoids. At the same time, eggs are a moderate energy source, show great culinary potential, and are widely available at an affordable price [28,29,30,31]. In addition, they are a vitamin-rich food containing all vitamins except vitamin C. Besides the mentioned folates, high amounts of vitamin A, D, E, K, B1, B2, B5, B6 and B12 can be found in egg yolk, while egg white has significant concentrations of vitamins B2, B3, and B5. Two eggs per day can cover from 10% up to 30% of the human demand for vitamins. Eggs are also one of the major sources of choline, concentrated in egg yolk (680 mg/100 g) [31]. Due to such a rich composition, eggs cannot be missing in an everyday diet of, primarily, children, pregnant women, breastfeeding mothers, and the elderly.

The quality of eggs is confirmed by numerous analyses performed to determine their freshness, microbiological safety, nutritional composition, contents of harmful substances (like heavy metal pollution), as well as technological and sensorial properties to suit both the food industry requirements and consumer demands. One way to increase the nutritional value of eggs and at the same time to make them more attractive to the consumers, is their enrichment via poultry feed manipulation. One of the examples is egg biofortification with such biologically-active antioxidants like omega-3 fatty acids and carotenoids [28]. Other examples include hen diet modification by oil addition or hen feeding with feedstuffs having a high content of unsaturated fatty acids (fish, chia, flaxseeds, olive or soy oil). Larger dietary supply of some trace minerals, like selenium or iodine, to hen can also increase their content in eggs. By hen feed mixture modification, eggs can also be enriched in both, lipophilic (A, D, E, K) and water-soluble vitamins, including folates (see Reference [31] for a review).

The results from our previous studies [32,33] confirmed high folate content in animal liver and satisfactory stability of certain folate vitamers under various cooking methods in chicken liver. Limited information on folate content in eggs, which are reported to have the potential to improve folate supply, have prompted us to deliver basic knowledge in this field. While the previous research concerned mainly eggs from laying hen farms with known [34] and specially developed feeding [21], the aim of this study was to evaluate the folate content in eggs from different rearing systems (organic, free barn, barn, and cage) obtained as they would be bought by consumers from the local market on their daily routine. A second objective was to present the distribution of different folate vitamers and their stability under cooking because most of the available data provide only the total folate content. It is believed that such data is of particular importance since information on various chemical folate derivatives is essential to predict their stability in different food products and their bioavailability. Finally, the results of the work are expected to provide important and practical information for consumers on the contribution of eggs from different farming systems to the daily folate intake. Additionally, due to the importance of eggs sensory characteristics for consumers, the color parameters of tested egg yolks were also evaluated.

## 2. Results and Discussion

Eggs are eaten worldwide and there is consumption growing globally. The European Union, with its egg production for consumption purposes peaking to 7.5 million tons in 2016, is the second-largest egg producer after China. In 2016, 7.5 million tons of eggs were produced in the EU for consumption purposes. The European biggest producers include Germany, France, Spain, Italy, Poland, the Netherlands, and the United Kingdom. The egg market cannot remain unchanged. It needs to adapt to the changing and growing expectations of consumers. The increasing awareness of animal welfare coupled with a trend towards more environmentally friendly and healthier food, have triggered significant changes in the egg market in terms of the rearing conditions of raising hens and egg nutrient content [35,36]. According to recent studies, the price is still the most influential factor in consumers’ egg-purchasing decision [35,36,37]. Therefore, eggs from the cage system are the most popular on the market. However, a successive increase in the interest in more expensive eggs from the free-range system is observed. Żakowska-Biemans’s study results [35] are consistent with the observed public growing concern for animal welfare, showing the farming system next to the price, to have the most significant mean relative importance in shaping consumers’ preferences. Free range eggs are perceived by consumers as traditional food of higher quality with hens having access to a chicken run and administered a more varied diet.

According to the study into the Polish consumer preferences regarding information on the farming system and nutritional enhancement of eggs [35], free range eggs were preferred over organic ones. Eggs from the free-range system had the highest relative importance for consumers even though organic eggs are produced under much stricter animal welfare standards according to the Regulation (EU) 20118/848 of the European Parliament and of the Council [38]. In ecological breeding, the birds are provided with the same conditions as in the case of free-range breeding. The main difference lies in the type of feed used, which in this case must be organic. Nevertheless, the free-range claims generate more market opportunities than the organic ones. Less preferred were barn eggs, and consumers were aware of the difference between the free range and barn rearing systems and favored access to the outdoors [35].

However, the effect of the farming system on egg folate content has not been sufficiently explored. There are no literature data regarding the distribution of different folate vitamers in eggs available on store shelves, in retail chains, and local markets. Based on an interview, eggs from different manufacturers representing four different rearing systems were selected at a similar time from the laying. The selection of eggs that are at the consumer’s reach on the daily basis took into account potential folate storage and handling losses or interconversions in fresh products and provided more reliable information of its nutritional composition. In the performed pilot study, no folates were found in the egg white, which is in agreement with previous studies by Sherwood et al. [39], who reported no folates when using a radioisotope dilution method and by Strandler et al. [34] who used a microbiological assay. The HPLC analysis of the egg yolk allowed identifying two folate forms, i.e., 5-CH_3_-H_4_folate and 10-HCO-folic acid. The same folate vitamins were detected in earlier studies [34,40,41] using liquid chromatography or microbiological methods. Strandler et al. [34] pointed out that it was of special interest to determine 10-HCO-folic acid-the oxidation product of 10-HCO-H_4_folate- which is not a naturally occurring form, but has vitamin activity. Similarly to the cited studies, other folate forms, like H_4_folate, 5-HCO-H_4_folate, and folic acid, were not detected. Folate content in raw samples of whole hen eggs from four different farming systems is presented on fresh weight (FW) basis in Table 1.

In all analyzed eggs, the major folate form was 5-CH_3_-H_4_folate, which accounted for more than 90% of total folates. Methyl folate form content ranged from 63.5 µg/100 g in free range egg sample to 143 µg/100 g in organic egg sample (see the supplementary materials). The lowest formyl form content (3.0 µg/100 g) was found in eggs from the caged system while the highest (9.5 µg/100 g) in the free-range sample. All analyzed egg samples had a high total folate content. The highest mean total folate content (113.8 µg/100 g) was found in organic eggs and was significantly different (*p* < 0.05) from contents of these vitamins in eggs from free range, barn and caged systems. The lowest mean folate contents were determined in eggs from the caged farming system (78.5 µg/100 g). However, these eggs were not found to differ significantly (*p* < 0.05) in folate content compared with the free range and barn ones. The results obtained for organic eggs correspond well to the values presented for eggs of unknown origin by Soongsongkiat et al. (117 µg/100 g) [42] and Yon et al. [43] (114 µg/100 g). Organic eggs from our study showed folate levels (93.7–142.8) comparable those found in folate-enriched free-range eggs reported by Altic et al. [21] (123.2 µg/100 g). These authors found the total folate content in un-enriched barn and un-enriched free-range eggs at the much lower levels, i.e., 41.4 and 65.6 µg/100 g, respectively, compared to our results for eggs from the same farming system. The difference observed in folate content between eggs from these two rearing systems was explained by Altic et al. [21] by the fact that free range hens have access to the fresh forage and this can provide an extra folate source to their daily feed. Barn hens are only fed with processed feed, probably low in folates. Therefore, the significantly higher folate contents obtained in our study in organic eggs can be explained by more restrictive requirements for the ecological method of farming and feed ingredients, which all together result in a higher folate status in a laying hen diet. In organic farming, the period of covering the soil with plants eaten by the hens is maximally extended by frequent sowing. The use of artificial fertilizers and pesticides is prohibited. The soil is fertilized only with natural products like compost or manure. Chickens have access to green pastures and fodder produced only using the organic methods. The group of organic farms is under constant veterinary supervision. Lower than presented in our study folate levels are provided in different food databases, but for eggs from the unspecified rearing system, i.e., 65 µg/100 g for Poland [44], 71 µg/100 g for the USA [45], and 21–69 µg/100 g for Denmark, Finland, Norway, and the UK [34]. Higher values are given in the Swedish Food Database; being 83.6 µg/100 g for organic egg and 85.8 µg/100 g for egg of unspecified farming system [46]. In fact, many factors can be responsible for folate content and egg quality in general, including genetic factors like breed and line of laying hens, which can affect shell size and color together with nutritional values. Also the type of rearing, housing conditions, and feeding system, like feed mixture composition and access to free range, have a substantial impact on egg composition. Even age of hens influence egg weight and shell quality as well as egg storage time and conditions [47,48,49,50,51,52,53,54,55]. However, the difference in folate content, provided by various sources, may also result from various methods of their determination. We reviewed problems related to the food folate analysis in our previous work [56], indicating critical points in extraction procedures as well as in methods used for folate detection and quantification. Difficulties in analyzing folates together with scarce information on method validation parameters, and deficient certified reference materials, caused a broad lack of folate data in food composition tables.

Table 2 presents the estimated folate intake per capita for the Polish population, from one raw egg (average weight 60 g) from various types of farming. Folate intake was estimated based on the Recommended Daily Allowance (RDA) presented in the Nutrition Standards for the Polish Population of the Institute of Food and Nutrition [57]. Folates absorption in the digestive tract is limited. It is assumed that the effective absorption of dietary folate does not exceed 50%, while that of folic acid from a dietary supplement can reach up to 100%. Given the differences in the bioavailability of folate from different sources, the mean total folate content in eggs is expressed in µg of dietary folate equivalent (DFE), where 1 µg DFE is equal to 1 µg dietary folate = 0.6 µg of folic acid from fortified foods or dietary supplements consumed with foods = 0.5 µg of folic acid from dietary supplements taken on an empty stomach [58]. The demand for folates depends on age and physiological condition, being higher for pregnant and lactating women. Women of the childbearing age are recommended to supplement their diet with folic acid in a dose of 0.4 mg/day, especially in the months before pregnancy and in the first trimester.

Considering the recommended daily allowance (RDA), the studied eggs can be a significant folate source in an everyday diet. One organic egg in the diet of the youngest children can cover even up to half of the RDA for folates, whereas in the older children group with a higher folate demand, it can cover from 16% (caged eggs) to 23% (organic eggs) of RDA. The selection of folate-rich eggs, like the organic ones, can increase the coverage of daily demand for folates in the group of the youngest teenagers up to 23–27%. In the case of adults, the RDA for folates is covered in the lowest percentage (12%) by caged eggs and in the highest percentage (17%) by the organic ones. However, in the adult population group, the importance of eggs in meeting the daily requirement for folates is even greater considering the higher frequency of egg consumption in this group, which is consistent with studies confirming the safe consumption of up to 14 eggs per week without a significant effect on the LDL cholesterol content [27]. In the diet of pregnant and breast-feeding women with the highest folate demands, the eggs offer various RDA coverage rates depending on the rearing system, i.e., from 8% for caged, 9% for barn and free range, to 11% for organic eggs.

Natural dietary folates are one of the most unstable vitamins. Individual folate vitamers show markedly different stabilities [7]. They degrade under the influence of temperature, oxygen, and sunlight [59] and, therefore, can be prone to losses during pre-processing, various cooking treatments, and storage. Folate retention has recently been studied in most common plant-derived sources, such as legumes, grain products, green leafy and other vegetables. Significant folate losses after cooking using different methods, such as steaming, blanching, boiling, microwaving, canning, or sous-vide cooking, reaching even up to 70–95% of initial folate content have been reported, both under domestic conditions and on an industrial scale [3,7,23,25,60,61,62]. The current results showed a considerable difference in the stability of the two folate vitamers, 5-CH_3_-H_4_folate and 10-HCO-folic acid, during egg hard-boiling (Table 3). The retention of a more stable methyl form was found to peak to 93% for organic, 86% for free range, and 98% for barn and caged eggs. In all analyzed eggs, the 5-CH_3_-H_4_folate retention was not lower than 77%. Meanwhile, 10-HCO-folic acid losses were high in all analyzed groups of eggs-up to 44.8% in organic, 43.4% in free range, 52.1% in barn, and 44.5% in caged eggs. In terms of the total folate content, the minimal losses were observed for caged (4.7–9.3%) and barn eggs (4.3–12.0%). Slightly higher losses were shown for eggs from organic farming (9.1–14.3%). In the group of free-range eggs, the losses exceeded 20% in two eggs. Although the literature provides scarce data on folate stability in eggs, previous reports– in agreement with the current study-showed folates in yolk from conventional eggs to be fairly stable following boiling, with losses not exceeding 10% [34,43]. Soongsongkiat et al. [42] demonstrated a much higher loss of 39%. In turn Altic et al. [21] reported that cooking folate-enriched eggs had no significant effect on folate stability. In turn, Seyoum et al. [63] explained the greater folate stability in eggs, compared to for instance plant-derived food, with the content of their antioxidants, and particularly of cysteine which presence may result in greater folate retention. Similarly, in our previous study on folate stability, high folate retention was observed after steaming, grilling or sous-vide cooking animal liver known to have a high content of cysteine [33].

**Figure 2:**
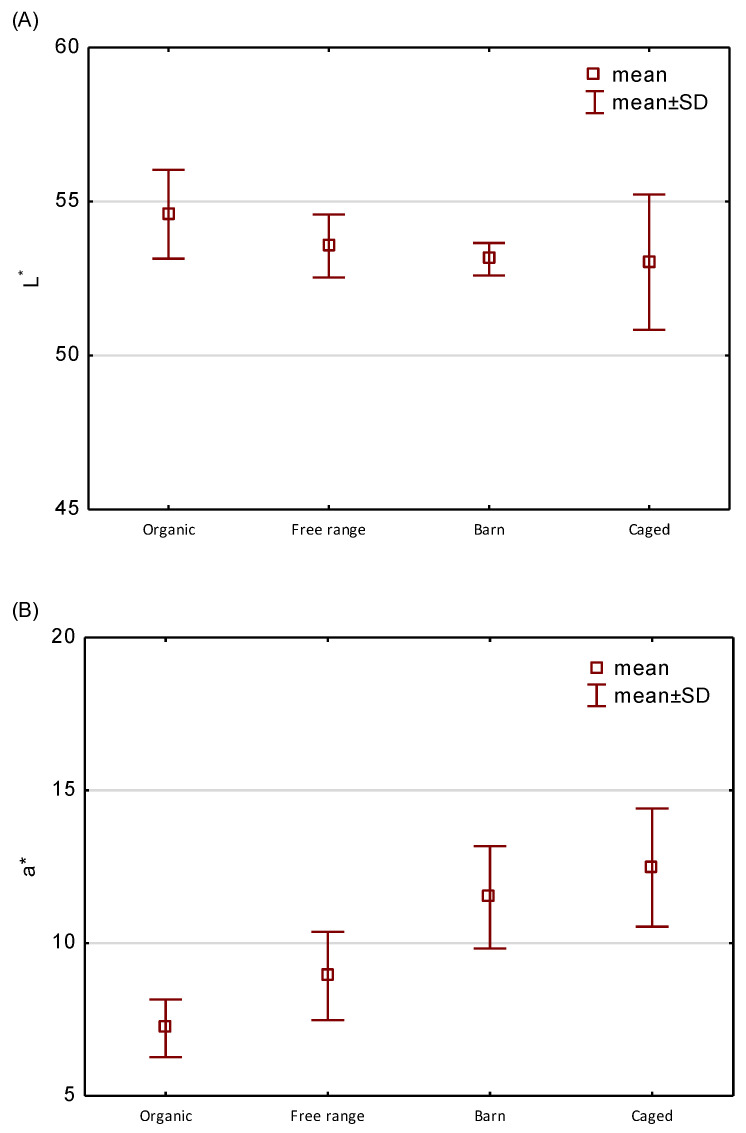

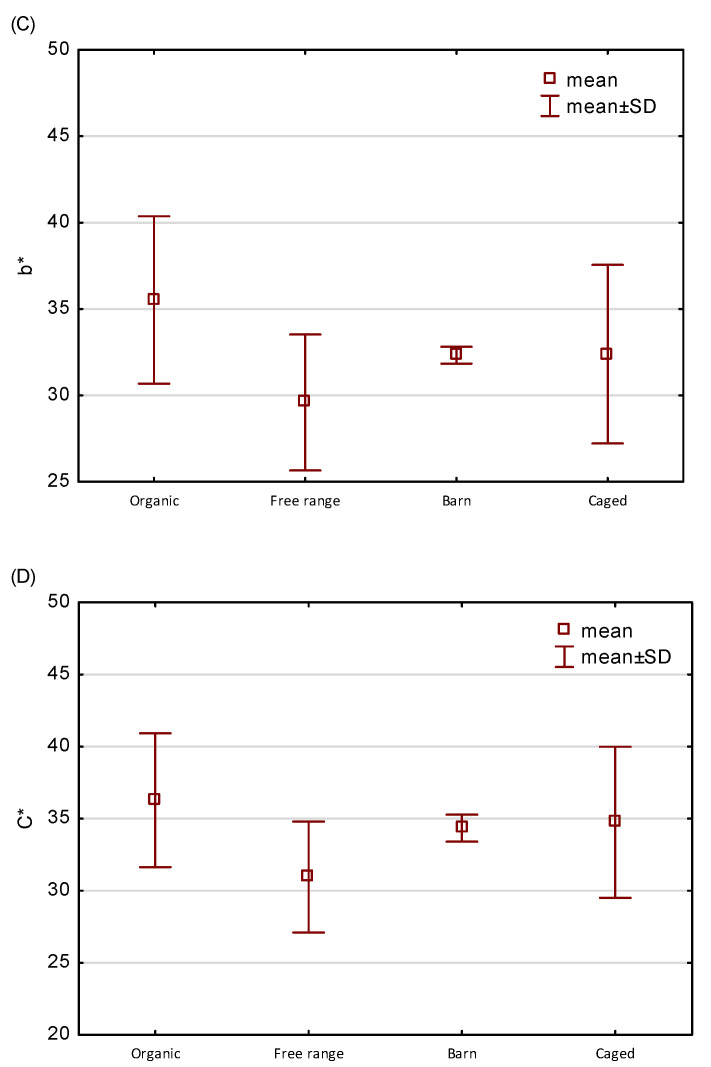
The effect of the farming system on the yolk color parameters (**A**) L*, (**B**) a*, (**C**) b*, and (**D**) C*.

Besides the nutritional composition of eggs, their sensory characteristics, including yolk color, are no less important. The results of surveys conducted in the last decade in a number of European countries have confirmed that yolk color intensity is an important indicator of its quality to consumers [64]. The effect of housing system on yolk color was confirmed in many studies [65]. Figure 2 shows the values of color parameters L*, a*, b*, and chroma (C*) of yolks of eggs from different rearing system. The lightness (L*) of egg yolks from organic (54.59) and free range (53.56) farming differed significantly (*p* < 0.05) from that of barn (53.13) and caged (53.03) eggs. The lowest value of redness (a*) parameter was determined for organic eggs (7.2) and differed significantly (*p* <0.05) from the other samples. The highest a* value was found in egg yolk from cage farming (12.5). The paler yolk color of organic eggs may be due to the fact that coloring feed additives are prohibited in organic production [66]. Regarding yolk yellowness (b*), there were no significant differences (*p* <0.05) in any of the analyzed egg groups, which proves that hens with their free access to green forage containing certain pigments, always produce eggs with aceptable yolk color [67]. The color chroma (C*) of organic egg yolks reached the highest value (36.2), being significantly (*p* <0.05) different from other eggs. Although consumer perception of egg yolk color is generally linked to the geographical location, culture and traditions, it is true that consumers in most parts of the world prefer deeply hued yolks [68] with the most preferred yolk color ranging from golden yellow to orange [67]. However, our study showed that organic eggs should not be discriminated because of lighter and less red color, as they may contain more bioactive substances, e.g., folates.

## 3. Materials and Methods

### 3.1. Samples

Eggs of hens from four different systems of farming (organic, free range, barn, and cage) were purchased at the local market of Olsztyn area including shops, delicatessen, and retail chains since May till August 2020. Each type of egg, from different farming system, was purchased from three different manufacturers (*n* = 12). For every type of egg, from every producer, samples of raw eggs (*n* = 6) and boiled ones (*n* = 6) were prepared for folate content determination. The samples of raw whole eggs were weighed without the shell and blended in a vacuum blender (Bosch; Gerlingen, Germany). Other eggs were cooked in an egg cooker (KEB 350, KOENIC; Ingolstadt, Germany) with the hardness adjustment set to the highest level, corresponding to hard-boiled eggs, and then allowed to cool in cold water, peeled, weighed, and blended.

### 3.2. Chemicals, Enzymes, and Standards

Water was purified in the Mili-Q system (Millipore; Vienna, Austria), acetonitrile was of HPLC grade, while other chemicals used were of analytical grade. α-Amylase [E.C.3.2.1.1] and protease [E.C.3.4.24.31] were obtained from Sigma Aldrich, while fresh rat plasma, used as a folate conjugase source, was purchased from Europa Bioproducts Ltd. (Cambridge, UK) and prepared according to Patring et al. [69]. Briefly, 50 mL of fresh plasma were dialyzed under stirring in 0.05 M phosphate buffer, pH 6.1, with 0.1% (*v/v*) 2-mercaptoethanol at 4 °C/12 h to remove endogenous folate. The buffer was changed three times during the dialysis. No presence of endogenous folate was confirmed in the HPLC analysis. Rat plasma was stored at 70 °C, for no longer than 3 months. The enzyme activity was checked using pteroyltri-L-glutamic acid (PteGlu_3_) according to Vahteristo et al. [70].

Folate standards: folic acid, 5-methyltetrahydrofolate (5-CH_3_-H_4_folate), 5-formyltetrahydrofolate (5-HCO-H_4_folate), and tetrahydrofolate (H_4_folate), were obtained from Sigma Aldrich (St. Louis, MO, USA); whereas 10-formyl folic acid (10-HCO-folic acid) and 5,10-methenyltetrahydrofolate (5,10-CH^+^-H_4_folate) were obtained from Schircks Laboratories (Jona, Switzerland). All standards were prepared as described by Konings [71]. 10-Formyldihydrofolate (10-HCO-H_2_folate) was obtained from 5,10-CH^+^-H_4_folate according to Pfeiffer et al. [72].

### 3.3. Sample Preparation

The content of folate vitamers was analyzed in triplicate using the sample pretreatment method described by Czarnowska-Kujawska et al. [32]. Briefly, 1 g (accurate to 0.001 g) of egg sample was inserted into a 30-mL PPCO Oak Ridge PPCO centrifuge tube (Nalgene; Rochester, NY, USA). Then, 20 mL of an extraction buffer (0.1 M phosphate buffer, pH 6.1, with 1% (*w*/*v*) sodium ascorbate and 0.1% (*v*/*v*) 2-mercaptoethanol) were added. Samples were homogenized (T 25 IKA WERKE Ultra-Turax homogenizer T 25 IKA WERKE; Staufen, Germany) for 1 min (21,500 rpm), and then transferred into a boiling water bath, heated therein for 15 min, shaken three times (2500 rpm/10 s Vortex 4 basic IKA Vortex 4 basic), and cooled in ice. 1 mL of the α-amylase solution (20 mg/mL) and 0.25 mL of rat plasma conjugase were added to each sample, and the samples were incubated at 37 °C for 4 h (POL-EKO; Rybnik, Poland). One hour later another enzyme, protease (4 mg/mL), was added. During incubation, samples were under mild stirring (magnetic stirrer). Afterward, they were heated in a boiling water bath for 15 min, then cooled in ice, and finally centrifuged twice at 12,000 rpm/4°C/20 min (MPW-350R, company; Warsaw, Poland). Each time, supernatants were collected to 50-mL amber volumetric flasks, which were filled up with the extraction buffer. The extract was filtered through the filter paper into amber glass bottles, flushed with nitrogen, and stored at −70 °C until the HPLC analysis.

Prior to the HPLC analysis, the samples were purified using Solid Phase Extraction (SPE) on Strong Anion Exchange (SAX) Bakerbond SPE JT cartridges [3 mL × 500 mg Solid Phase Extraction Column, PP (polypropylene), Quaternary Amine (N^+^) Anion Exchange; Philipsburg, MT, USA] as described by Jastrebova et al. [73]. Briefly, 6 mL of the sample were inserted onto the SAX column pre-conditioned with methanol and water and eluted with 4 mL of the elution buffer (0.1 M sodium acetate containing 10% (*w*/*v*) sodium chloride and 0.1% (*v*/*v*) 2-mercaptoethanol).

### 3.4. Folate Quantification

The chromatographic separation of folates was carried out according to Hefni et al. [74] using the HPLC system (Shimadzu Nexera-i LC-2040 C plus; Shimadzu Co.; Kyoto, Japan) and the C18 LC column (150 × 4.6 mm, 3 µm, Luna 100Å; Phenomenex; Torrance, CA, USA). The total separation time was 42 min. Folates were separated under binary gradient elution conditions, with 30 mM phosphoric acid buffer (pH 2.3) and acetonitrile used as the mobile phases. The gradient started at 6% (*v*/*v*) acetonitrile maintained isocratically for the first 5 min, then raised linearly to 25% within 20 min. The flow rate was set at 0.4 mL/min and injection volume at 20 µL. The temperature of the column was 25°C. Peaks were identified based on standard retention times.

Quantification of the identified individual folate vitamers was based on fluorescence (290 nm excitation and 360 nm emission for 5-CH_3_-H_4_folate, 5-HCO-H_4_folate and H_4_folate; 360 nm excitation and 460 nm emission for 10-HCO-folic acid) and UV detection (290 nm for folic acid and 10-HCO-H_2_folate) using the external multilevel (*n* = 8) calibration curves. Limits of detection for folates were: 0.2 ng/mL for 5-CH_3_-H_4_folate, 0.8 ng/mL for 5-HCO-H_4_folate, 0.3 ng/mL for H_4_folate, 0.4 ng/mL for 10-HCO-folic acid, 1.1 ng/mL for folic acid and 0.6 ng/mL for 10-HCO-H_2_folate. The linearity range for identified in egg samples two folate vitamers were: 0.4–140 ng/mL for 5-CH_3_-H_4_folate (the correlation coefficient > 0.9998) and 1.6–120 ng/mL for 10-HCO-folic acid (the correlation coefficient > 0.9993). The variation obtained between the replicates for each analyzed sample was lower than 10%. The repeatability of the analytical procedure was checked on different extraction days using raw and boiled egg samples. Recovery tests were performed by adding known amounts of 5-CH_3_-H_4_folate and 10-HCO-folic acid before the extraction to both raw and boiled eggs. Mean recovery (*n* = 4) was 91% ± 6 for 5-CH_3_-H_4_PteGlu and 87% ± 8 for 10-HCO folic acid.

The results of folate vitamer content determination in egg samples are based on the fresh weight (FW) and presented as means with standard deviations from triplicates. The total folate content is the sum of 5-CH_3_-H_4_folate and 10-HCO-folic acid contents expressed as folic acid content using a molar absorption coefficient given by Blakely [75]. Differences in the mean total folate content in eggs from different farming systems were compared using the Duncan multiple range test, with the significance level at *p* < 0.05. The statistical analysis was carried out using the Statistica software version 10.0 (StatSoft; Cracow, Poland).

### 3.5. Egg Yolk Color Analysis

The egg was cracked and its contents were placed in a Petri dish. Yolk color was determined using a CR-400 spectrophotometer (Konica Minolta; Osaka, Japan) with a measurement port of 8 mm in diameter. All measurements were conducted at three places of the whole egg (*n* = 3), i.e., one at the center and two on the side, at room temperature (20 °C ± 2 °C). The instrument was calibrated against a standard light white reference tile (CR-400 with Y = 89.3, x = 0.3159, y = 0.3225), and the measurements were conducted under standard illuminant D65 and a 2° standard observer angle. The color parameters: L*—lightness (− black, + white), a*—red/greenness (− green, + red), b*—blue/yellowness (− blue, + yellow), were determined and results were expressed in accordance to the CIELab color system. The color chroma (parameter C*) was calculated according to the following formula [76]:

(1) $$C^{*}=\sqrt{a^{*2}+b^{*2}}$$

One-way analysis of variance was used to show a significant influence of the farming system on the yolk color parameters. Duncan’s post-hoc test was used to test which pairs of means differ at the significance level of *p* < 0.05. Statistical analysis was carried out using Statistica 13.3 TIBCO Software Inc., 2020.

## 4. Conclusions

Folate bioavailability from natural sources is often limited compared with that of folic acid from dietary supplements of fortified foods. Thus, representative food composition data for folate vitamers in various foods, including eggs, are needed to evaluate the adequacy of the population’s folate intake. In our study, we described the distribution of two folate vitamers, 5-methyltetrahydrofolate and 10-formylfolic acid, present in egg yolk, showing the methyl form to be the dominant and more stable one.

According to our findings, the farming system may affect folate content in eggs. All eggs were found to be good folate sources, with the organic ones to be the most abundant in folates. They can significantly contribute to the coverage of the daily demand for folates in different population groups. The contribution of a boiled egg to daily folate demand coverage would still be considerable due to the minor folate loss during boiling. Organic egg folate potential inscribes well into the general trend of paying more attention to the origin of the eggs and rearing systems of hens by modern consumers. In turn, the visual evaluation of organic egg yolk-being paler than consumers may expect-can confusingly produce a feeling of its lower nutritional value.

## Figures and Tables

**Table 1:** Folate content in raw hen eggs from different farming systems (µg/100 g FW).

Farming System	5-CH_3_-H_4_Folate		10-HCO-Folic Acid	Total Folates (Sum as Folic Acid)	Mean Total Folate Content as Folic Acid
Organic	1 ^1^	101.4 ± 4.7 ^2^	7.6 ± 0.3	104.9 ± 4.6	113.8 ^a3^
	2	91.8 ± 0.5	5.5 ± 0.1	93.7 ± 0.5	
	3	143.0 ± 1.1	5.1 ± 0.4	142.8 ± 1.3	
Free range	1	88.0 ± 2.5	9.5 ± 0.5	93.8 ± 2.1	85.5 ^b^
	2	92.3 ± 4.9	8.2 ± 0.2	96.7 ± 4.7	
	3	63.5 ± 2.8	5.1 ± 0.5	66.1 ± 2.5	
Barn	1	86.4 ± 4.2	5.7 ± 0.2	88.6 ± 4.2	88.5 ^b^
	2	92.3 ± 7.8	3.5 ± 0.3	92.5 ± 8.8	
	3	82.9 ± 1.0	4.7 ± 0.4	84.4 ± 1.1	
Caged	1	78.4 ± 2.1	3.0 ± 0.2	78.4 ± 1.9	78.5 ^b^
	2	75.2 ± 1.6	4.6 ± 0.2	76.9 ± 1.4	
	3	79.1 ± 2.3	4.2 ±.0.1	80.3 ± 3.2	

^1^ Each farming system was represented by three producers; ^2^ The results are presented as the mean of three replicates ± standard deviation; ^3^ concerns the explanation of lowercase letters a and b, means in the column with the same letter are not significantly different at *P* < 0.05.

**Table 2:** Coverage of the daily demand for folates after eating one egg (60 g) from different farming systems.

		Group, Gender, Age, Years				
		Children 1–9	Teenage Boys 10–18	Teenage Girls 10–18	Men and Women ≥ 19	Pregnant and Breast-Feeding Women
		µg Folate Equivalent/Person/Day				
Farming System	RDA	150–300	250–330	300–400	400	600
Organic	Mean (µg/60g)	68.3				
	DDC %	23–46	21–27	17–23	17	11
Free range	Mean (µg/60g)	51.3				
	DDC %	17–34	16–21	13–17	13	9
Barn	Mean (µg/60g)	53.1				
	DDC %	18–35	16–21	13–18	13	9
Caged	Mean (µg/60g)	47.1				
	DDC %	16–31	14–19	12–16	12	8

Source: own study based on Jarosz et al. [57]. Abbreviations: RDA—Recommended Daily Allowance, DDC—Daily Demand Coverage.

**Table 3:** Folate losses after cooking (%) in hen eggs from different farming systems.

Farming System		5-CH_3_-H_4_ Folate (%)	10-HCO-Folic Acid (%)	Total Folates (Sum as Folic Acid) (%)
Organic	1 ^1^	12.0 ^2^	25.8	13.0
	2	7.1	44.8	9.1
	3	13.3	44.7	14.3
Free Range	1	18.4	39.3	20.4
	2	22.6	43.4	24.2
	3	14.2	11.9	14.0
Barn	1	4.4	47.3	7.0
	2	11.2	34.6	12.0
	3	1.7	52.1	4.3
Caged	1	8.2	30.4	9.0
	2	2.5	41.5	4.7
	3	7.5	44.5	9.3

^1^ Each farming system was represented by three producers. ^2^ The results are presented as the mean of three replicates.

## Data Availability

Not applicable.

## Supplementary Materials

The following are available online. Figure S1. HPLC-UV chromatogram (290 nm) of folate derivatives—standard mixture. Figure S2. HPLC-FLD chromatogram (ex. 290 nm, em. 360 nm) of folate derivatives—standard mixture. Figure S3. HPLC-FLD chromatogram (ex. 360 nm, em. 460 nm) of folate derivatives-standard mixture. Figure S4. HPLC-FLD chromatogram (ex. 290 nm, em. 360 nm) of folate derivatives—organic egg sample. Figure S5. HPLC-FLD chromatogram (ex. 360 nm, em. 460 nm) of folate derivatives—organic egg sample.

## Sample Availability

Samples are available from the authors.
