# Supplementary material for: Folate Content and Yolk Color of Hen Eggs from Different Farming Systems
Source: Molecules. 2021 Feb 16;26(4):1034. doi: 10.3390/molecules26041034 (PMC7919826; doi:10.3390/molecules26041034)
Supplement: Supplementary file 1 [file molecules-26-01034-s001.pdf]

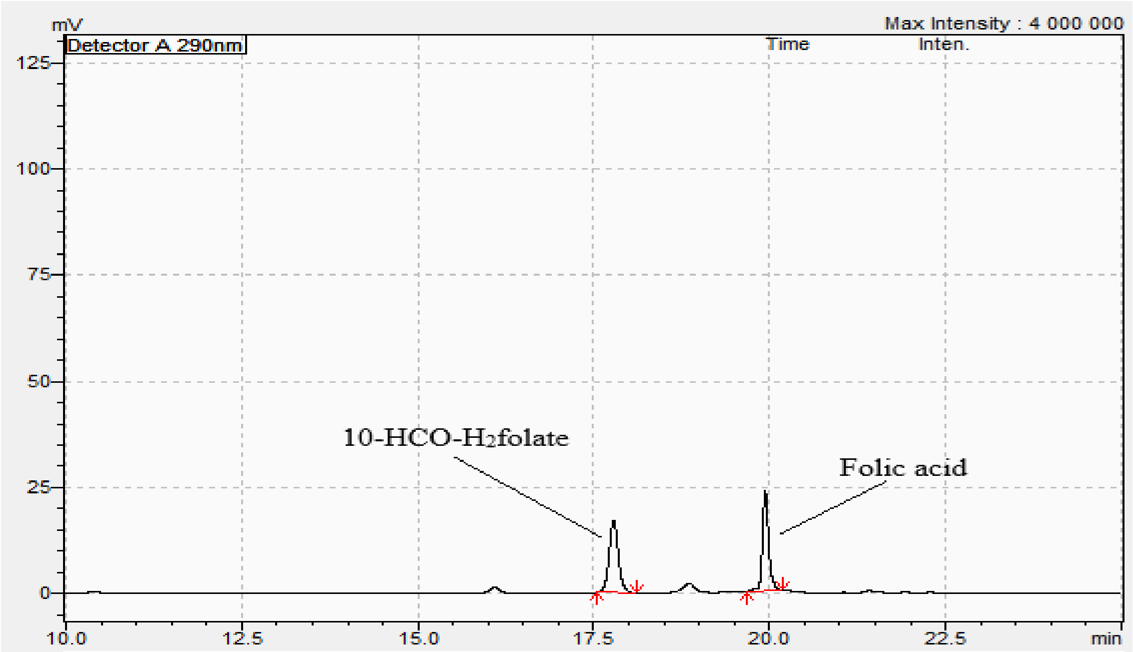

Fig. 1. HPLC-UV chromatogram (290 nm) of folate derivatives - standard mixture.

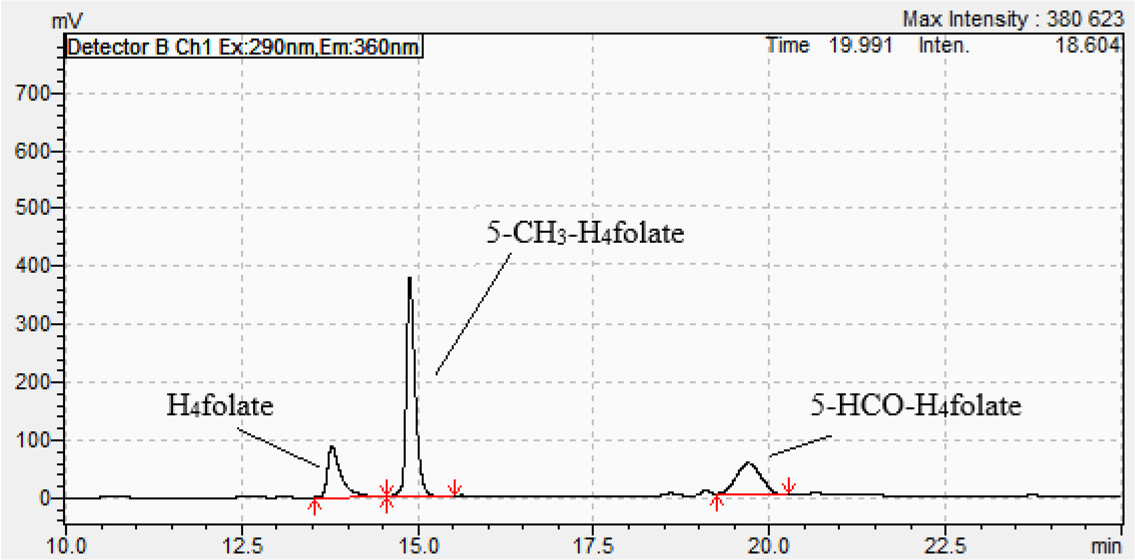

Fig. 2. HPLC-FLD chromatogram (ex. 290 nm, em. 360 nm) of folate derivatives - standard mixture.

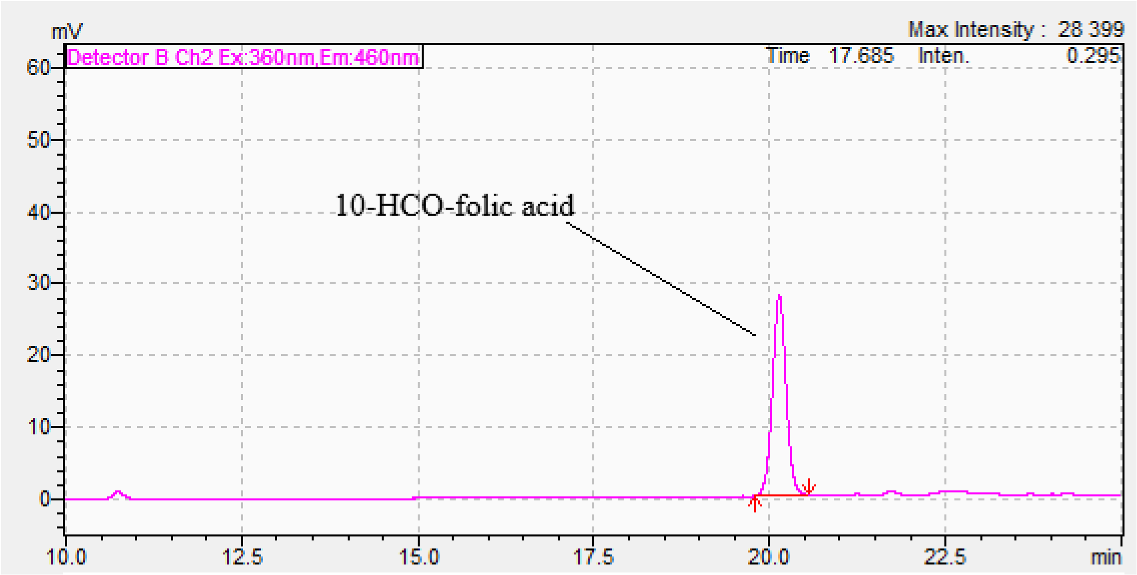

Fig. 3. HPLC-FLD chromatogram (ex. 360 nm, em. 460 nm) of folate derivatives - standard mixture.

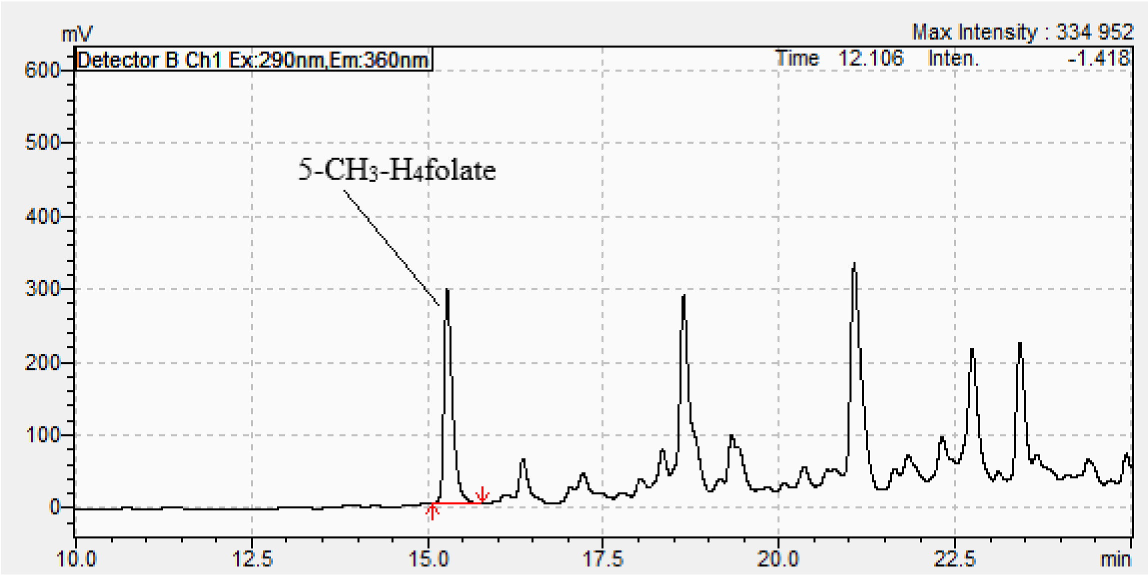

Fig. 4. HPLC-FLD chromatogram (ex. 290 nm, em. 360 nm) of folate derivatives – organic egg sample.

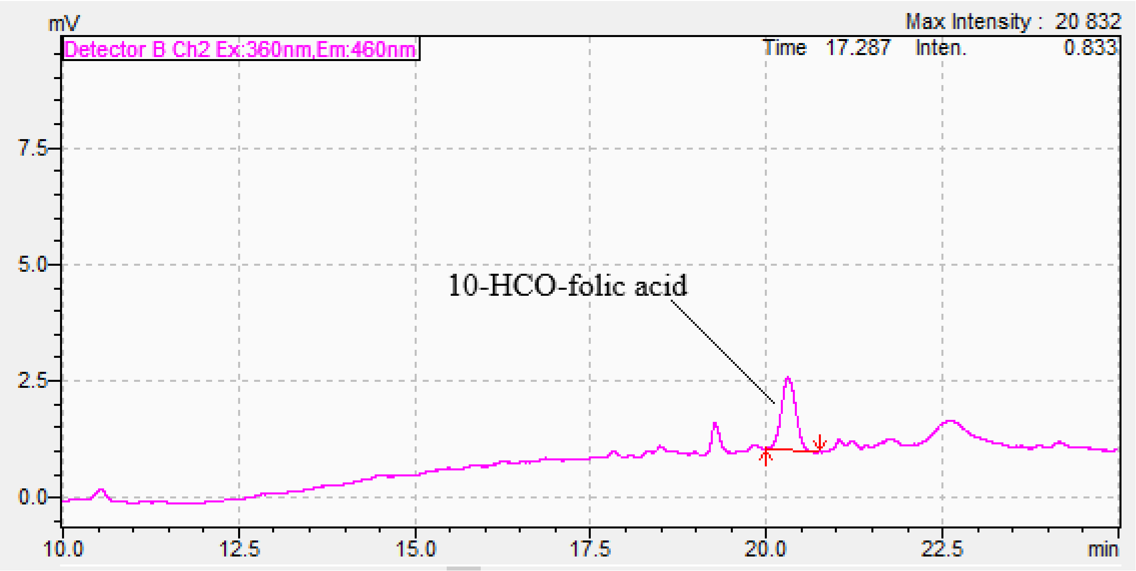

Fig. 5. HPLC-FLD chromatogram (ex. 360 nm, em. 460 nm) of folate derivatives – organic egg sample.
